# Fabrication and Characterization of MSQ Aerogel Coating on ePTFE Thin Films for Cable Sheaths

**DOI:** 10.3390/molecules24071246

**Published:** 2019-03-30

**Authors:** Xingzhong Guo, Shengchi Bai, Jiaqi Shan, Wei Lei, Ronghua Ding, Yun Zhang, Hui Yang

**Affiliations:** 1 School of Materials Science and Engineering, Zhejiang University, Hangzhou 310027, China; baishengchi@163.com (S.B.); 21626008@zju.edu.cn (J.S.); yanghui@zju.edu.cn (H.Y.); 2 Pan Asia Microvent Tech (Jiangsu) Corporation & Zhejiang University Micro-nano-porous Materials United Research Development Center, Changzhou 213100, China; leiwei@microvent.com.cn (W.L.); dingronghua@microvent.com.cn (R.D.); zhangyun@microvent.com.cn (Y.Z.)

**Keywords:** MSQ aerogel, ePTFE, aerogel coating, cable sheath

## Abstract

With methylsilsesquioxane (MSQ) aerogels synthesized by the sol-gel method as a raw material and Si-Ti sol as a binder, an alcohol-based aerogel slurry consisting of only MSQ aerogel and Si-Ti sol was prepared and coated on expanded polytetrafluoroethylene (ePTFE) to form an MSQ aerogel coating layer, followed by low-temperature heat treatment. The effect of Si-Ti sol content on the microstructure of the MSQ aerogel coating layer was investigated, and the properties of a typical MSQ aerogel-layer-coated ePTFE film were evaluated. The results show that Si-Ti sol has an important role in terms of film-forming capability, surface smoothness, flexibility, and powder dropping of the MSQ aerogel coating layer. With a Si-Ti sol of 10.5 wt.% content as a binder and after heat treatment at 170 °C for 30 min, the coated ePTFE flexible thin film with a layer thickness of 30 μm shows high uniformity, integrity, and electrical insulation properties, with an elongation at break decrease over 130%, a thermal conductivity of 0.1753 W/(m·K) at 25 °C, a dielectric constant of 16.5674, and a dielectric loss of 0.06369, which can be promisingly applied in cable sheaths.

## 1. Introduction

In modern society, cables have been applied extensively in electronic devices, enabling the transfer of electrical signals or power. Thus, it is vital for cables to survive and perform reliably, whether used in systems operating on land, in the ocean, in the air, or even in space. For durable cables, cable sheaths play a crucial role in protecting weak central cores, requesting stability, flexibility, and high electrical properties [1]. Many materials, such as polyurethane, polyethylene, polyimide, and fluoropolymers, are applied as cable sheaths. Expanded Polytetrafluoroethylene (ePTFE) [2,3,4,5,6,7] cable sheath material shows outstanding stability, mechanical, electrical, and thermal properties with low density, and has been regarded as a potential rival for high-performance cables. For high-performance ePTFE material, composites with other materials are a promising approach, such as CeO_2_ [8], SrTiO_3_ [9], and CaTiO_3_ [10]. Introduced inorganic materials, however, are harmful to flexibility and increase the density of the sheath, and the fabrication process is complex and high-cost, which is not suitable for a cable sheath.

Aerogels are randomly interconnected nanoscale clusters of metal oxides containing micro- and meso-porous networks, and thus exhibit unique physical properties, such as low density, optical transparency, a high surface area, high porosity, low thermal conductivity, a low refractive index, and a low dielectric constant [11,12,13,14]. In recent years, different kinds of aerogels have been investigated widely, including silica aerogel, zirconia aerogel [15,16,17,18], graphene aerogel [19,20,21], carbon aerogel [22,23], and cellulose aerogel [24,25,26,27]. Among these aerogels, silica aerogel was the first created (more than 80 years ago), but due to the low cost and outstanding properties, many extended applications have been developed for it, such as low-*k* materials, electrodes, thermal insulators, and transparent thermal insulators [11]. However, silica aerogels lack mechanical durability, which limits their application. To resolve this problem, organic–inorganic hybridization [28,29,30] using organotrialkoxysilanes, especially methyltrimethoxysilane (MTMS), as co-precursors with tetra alkoxysilane is a promising way to promote mechanical durability and flexibility. Methylsilsesquioxane (MSQ) aerogel is a kind of methyl hybrid SiO_2_ aerogel monolith synthesized via a sol-gel process [28,29]. Due to the methyl groups, the microstructure of MSQ aerogel tends to noncontiguous networks of bead chains rather than conventional three-dimensional space network structures [31]. This unique structure results in better flexibility, lipophilicity, and hydrophobicity [28,29]. However, for monolithic silica aerogels, the mechanical properties, such as elastic modulus and flexibility, are still not satisfactory and the fragile monolith may drop powder during transportation and application. Aerogel films show similar properties. Monolithic silica aerogel is suitable as a coating on other kinds of substrates, avoiding the drop of powder and promoting mechanical properties [32,33,34,35,36]. Traditionally, aerogel films were synthesized via coating precursor solutions onto substrates, especially glass, by dip coating, spin coating, spray coating, or other methods, before application and a supercritical drying process. Because of the low thickness, a solvent atmosphere was required, especially in spin coating [37,38,39]. It is obvious that these methods are costly and laborious, and not suitable for the fabrication of a flexible and even elastic film with an aerogel coating.

In this work, we firstly report an MSQ aerogel coating layer on ePTFE thin film for a cable sheath. MSQ aerogel was synthesized via a sol-gel process followed by ambient drying, showing a high specific surface area of 681.64 m^2^/g with an average pore diameter of 37 nm. The MSQ aerogel coating layer was fabricated on ePTFE thin film by a blade coating of alcohol-based aerogel slurry with MSQ aerogel powder as a raw material, Si-Ti sol as a binder, and ethanol as a solvent. The Si-Ti sol enhances the connection between MSQ aerogel powder and hydrophobic ePTFE film effectively to form a smooth aerogel film and eliminate the drop of MSQ powder. With 10.5 wt.% Si-Ti sol as binder, an MSQ aerogel coating layer on thin ePTFE film with a thickness of 30 μm exhibited high flexibility, uniformity, and integrity, with an elongation at break decrease, high thermal conductivity, and a low dielectric constant and dielectric loss. As described above, the uniform coating layer utilizing MSQ aerogel slurry is fabricated via a simple blade coating process, without harmful solvents or a costly high temperature process, which is suitable for a large-scale roll-to-roll process. Compared with hard and dense PTFE-ceramic material composite film, the aerogel layer-coated ePTFE film is flexible and lightweight with a similar dielectric property and a higher electrical insulation property, which implies that the aerogel-layer-coated ePTFE film has a wide range of applications.

## 2. Results

### 2.1. Characteristics of Monolithic MSQ Aerogel

**Figure 1:**
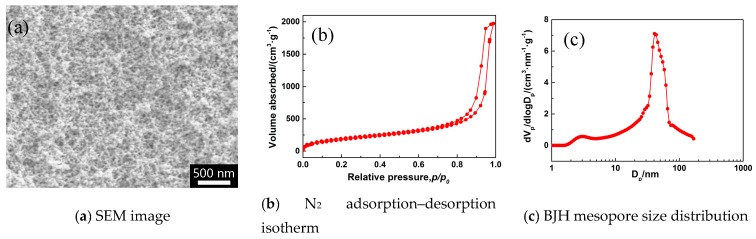
(**a**) SEM image of the synthesized methylsilsesquioxane (MSQ) aerogel and the N_2_ adsorption–desorption isotherm (**b**) and Barrett-Joyner-Halenda (BJH) mesopore size distribution (**c**) of the synthesized MSQ aerogel monolith.

The MSQ aerogel monolith was synthesized via a sol-gel process with MTMS as the procedure, hydrochloric acid (HCl) as catalyst, water and methanol as solvents, hexadecyltrimethylammonium chloride (CTAC) as a surfactant and template, propylene oxide (PO) as a gelation agent, and 2-propanol, hexamethyldisilane/n-heptane, and heptane for multistage solvent replacement followed by ambient drying. According to the previous report [28,31], the MSQ aerogel monolith shows a unique structure in that nanoparticles form a ‘‘string-of-pearls’’-type structure owing to the introduced Si-CH_3_ groups as shown in Figure 1a. The porous structure of the synthesized MSQ aerogel monolith is uniformly constructed by small spherical nanoparticles with an average diameter below 10 nm.

The pore structures of the as-prepared MSQ aerogel powder were further measured, and the N_2_ adsorption–desorption isotherm and BJH mesopore size distribution are shown in Figure 1. The N_2_ adsorption–desorption isotherm of the MSQ aerogel powder after ball milling belongs to type IV according to the classification of IUPAC, proving that synthesized MSQ aerogels have uniform ampulliform mesopores. As mentioned above, the skeletons of MSQ aerogels are formed by point-connected spherical nanoparticles rather than conventional three-dimensional space network structures, resulting in the unique ampulliform mesopores. Finally, the MSQ aerogel powder exhibits a high specific surface area of 681.64 m^2^/g with an average pore diameter of 37 nm.

### 2.2. Characteristics and Microstructure of the MSQ Aerogel Coating Layer on ePTFE Thin Film

**Figure 2:**
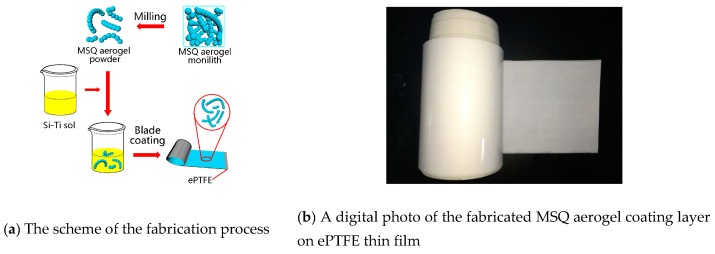
(**a**) The fabrication process for an MSQ aerogel coating layer on Polytetrafluoroethylene (ePTFE) thin film; (**b**) a digital photo of the fabricated large-scale MSQ aerogel coating on ePTFE thin film.

Fabricated monolithic MSQ aerogel shows a high porosity; however, for industrial application, the problems of dropped powder and poor mechanical properties and flexibility need to be solved. Thus, ePTFE film was chosen as a substrate, because it has great mechanical strength and flexibility. While ePTFE is chemically inert and hydrophobic, showing high stability, these properties also result in poor adhesion between MSQ aerogel and ePTFE. The precursor solution of MSQ aerogel can hardly gelatinize on the ePTFE film and form MSQ aerogel. The fabrication process for an MSQ aerogel coating layer on ePTFE film is shown in Figure 2a. Firstly, MSQ aerogel powder was fabricated using a milling process until the particle size was <5000 mesh. Then, the MSQ aerogel powder was mixed with Si-Ti sol and, after high-speed stirring for 30 min, an alcohol-based aerogel slurry was fabricated. Compared with other binders, such as polyvinyl alcohol (PVA) and Polyacrylic acid (PAA), the Si-Ti sol is more suitable for the hydrophobic ePTFE film. The slurry was poured onto the surface of ePTFE film. With the scraper sliding the film, a uniform MSQ aerogel film formed. The dried MSQ aerogel film was further heat-treated at 170 °C for 30 min to cure the binder and eliminate solvent. A digital photo of the fabricated MSQ aerogel coating layer on ePTFE thin film is shown in Figure 2b. The blade coating process is suitable for large-scale fabrication of an MSQ coating layer on ePTFE film with high uniformity and low costs.

**Figure 3:**
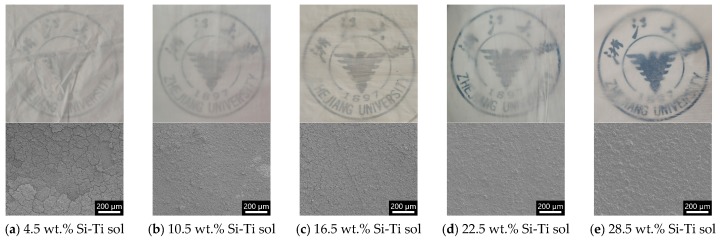
Digital photos and SEM images of the MSQ aerogel coating on ePTFE thin films with different Si-Ti sol contents. (**a**) 4.5 wt.%, (**b**) 10.5 wt.%, (**c**) 16.5 wt.%, (**d**) 22.5 wt.%, (**e**) 28.5 wt.%.

Figure 3 displays low-magnification SEM images and digital photos of an MSQ aerogel coating layer on ePTFE thin film with a different content of Si-Ti sol. Due to the low surface energy of ePTFE, the adhesion between MSQ aerogel and ePTFE film is poor. Thus, Si-Ti sol was chosen as an adhesive to bind MSQ aerogel powder and ePTFE thin film. MSQ aerogel was coated on the surface of ePTFE film via the blade coating method to form an MSQ aerogel layer. With the increase of the Si-Ti sol content, the cracks on the aerogel layer gradually decrease and the surface of the coating layer becomes smoother. With 4.5 wt.% Si-Ti sol, the MSQ aerogel layer cracks, resulting in high roughness and poor adhesion. Moreover, for the lower content of Si-Ti sol, the MSQ aerogel layer is white and flexible, according to the color of MSQ aerogel powder, but aerogel powders may easily drop from the MSQ aerogel coating layer. The color of the coating layer transfers from white to transparent and the dropping powder is eliminated, even bending or twisting with the increasing Si-Ti sol. However, the high content of Si-Ti sol is harmful to the flexibility of the cured Si-Ti sol, forming brittle ceramic materials. With the addition of the 16.5 wt.% Si-Ti sol, the ePTFE thin film coated with an MSQ aerogel layer is white and highly flexible with no powder dropping, demonstrating the best film-forming properties.

**Figure 4:**
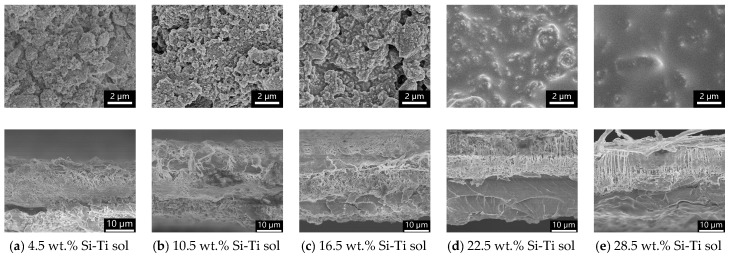
High-power and cross-section SEM images of MSQ aerogel coating on ePTFE thin films with different Si-Ti sol contents. (**a**) 4.5 wt.%, (**b**) 10.5 wt.%, (**c**) 16.5 wt.%, (**d**) 22.5 wt.%, (**e**) 28.5 wt.%

With the lower content of Si-Ti sol, the porous structure of MSQ aerogel is visible as shown in Figure 4a. With the increase in the content of Si-Ti sol, the pores of MSQ aerogel are gradually filled with cured Si-Ti sol, resulting in poor flexibility. The filled pores also result in an increase in the thermal conductivity and dielectric constant. As shown in the cross-section SEM images, the ePTFE thin film coated with an MSQ aerogel layer with different Si-Ti sol contents shows a similar thickness of about 30 μm. The cured Si-Ti sol forms a binding layer between ePTFE film and MSQ aerogel. During the fabrication process, some Si-Ti sol infiltrates into the surface of the porous ePTFE layer and cures in the heat treatment to form a compact layer. In other areas, Si-Ti sol covers the surface of MSQ aerogel powders and eventually binds the MSQ aerogel powders. With the lower content of Si-Ti sol, the thickness of the Si-Ti sol layer and the joining force between MSQ aerogel powders are insufficient, resulting in powder dropping and a rough surface. When the content of Si-Ti sol increases to 16.5 wt.%, MSQ aerogel powders are bound firmly with no powder dropping, even with repeated bending or twisting. They show high mechanical stability, and the porous structure of MSQ aerogel powder is preserved. The increasing Si-Ti sol also affects the density of the coating layer. For the 16.5 wt.% Si-Ti sol, the density is 0.18 g/cm^3^—even lower than that of monolith aerogel—and increases to 0.92 g/cm^3^ for 28.5 wt.% Si-Ti sol.

**Figure 5:**
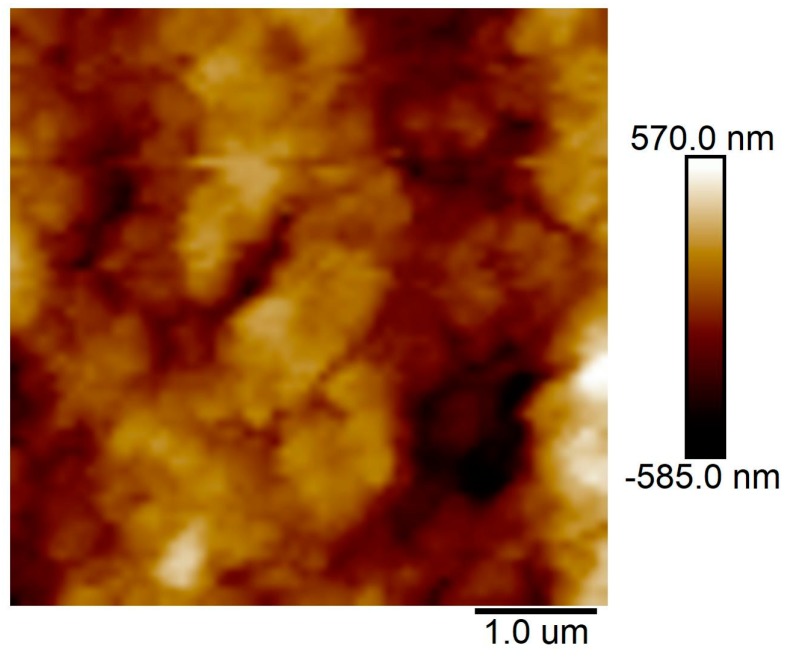
An AFM image of an MSQ aerogel coating layer on ePTFE thin film with 16.5 wt.% Si-Ti sol.

Figure 5 exhibits the AFM image of an MSQ aerogel coating layer on ePTFE film with 16.5 wt.% Si-Ti sol. The results show that the MSQ coating layer has a high level of roughness, and the root mean square (RMS) value reaches 558 nm. It can be seen from the AFM image that the MSQ aerogel layer has been formed by an accumulation of MSQ aerogel powders. The size of the MSQ aerogel particles is about 2 μm, according to the size of 5000 mesh. With the lower content of Si-Ti sol of 16.5 wt.%, MSQ aerogel powders form a uniform layer without cracks or dropped powder, but the pores of the MSQ aerogel are not completely filled by cured Si-Ti sol, resulting in a high RMS value.

### 2.3. Mechanical, Thermal, and Dielectric Properties of ePTFE Thin Film Coated with an MSQ Aerogel Coating Layer

**Figure 6:**
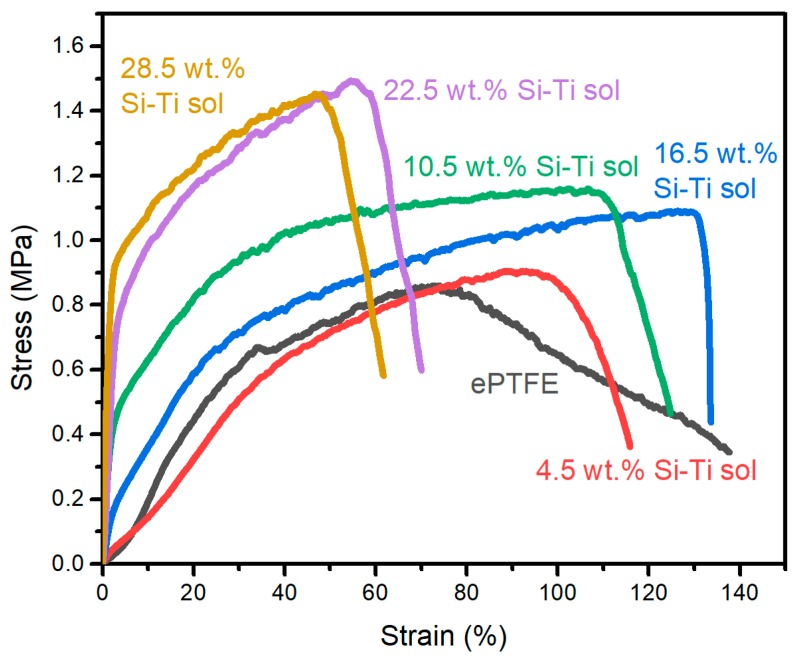
The stress-strain curve of MSQ aerogel layer coating on ePTFE thin films with different Si-Ti sol contents.

For application in cable sheaths, the mechanical and dielectric properties and the thermal conductivity of an MSQ aerogel coating on ePTFE thin film were tested. The pristine ePTFE film shows satisfactory mechanical properties. The mechanical properties after the ePTFE film was coated with an aerogel layer are shown as Figure 6. As mentioned above, for a lower content of Si-Ti sol, MSQ aerogel powders drop from the coating layer, which is not suitable for application in cable sheaths. The resulting mechanical property shows a slight increase in tensile strength from 0.86 MPa for the pristine ePTFE film to 0.91 MPa for the ePTFE film coated with 4.5 wt.% Si-Ti sol. The tensile strength of the coated ePTFE film gradually increases with increasing content of Si-Ti sol. However, a high content of Si-Ti sol (over 16.5 wt.%) would make the coated ePTFE film brittle and lower the mechanical properties, and the elongation at break decreases from over 130% for pristine ePTFE film to 70% (22.5 wt.% Si-Ti sol) and 61% (28.5 wt.% Si-Ti sol). In the MSQ coating layer, the content of Si-Ti sol significantly affects the mechanical properties. The Si-Ti sol forms a brittle and compact binding layer on the boundary between ePTFE film and the MSQ aerogel layer, which results in a change in mechanical properties. With the addition of a lower content of Si-Ti sol, the binding layer is thin and shows a slight effect. With the increase of Si-Ti sol content, the thickness of the binding layer increases, which reinforces the mechanical properties. However, when too much Si-Ti sol is added, the mechanical properties of the coated ePTFE film decrease, owing mainly to the brittle binding layer formed by the cured Si-Ti sol rather than the ePTFE film. This leads to the poor elongation at break and high tensile strength. The MSQ coating layer does not affect the flexibility of the ePTFE film, but enhances its mechanical properties, which is more suitable for application in cable sheaths.

**Figure 7:**
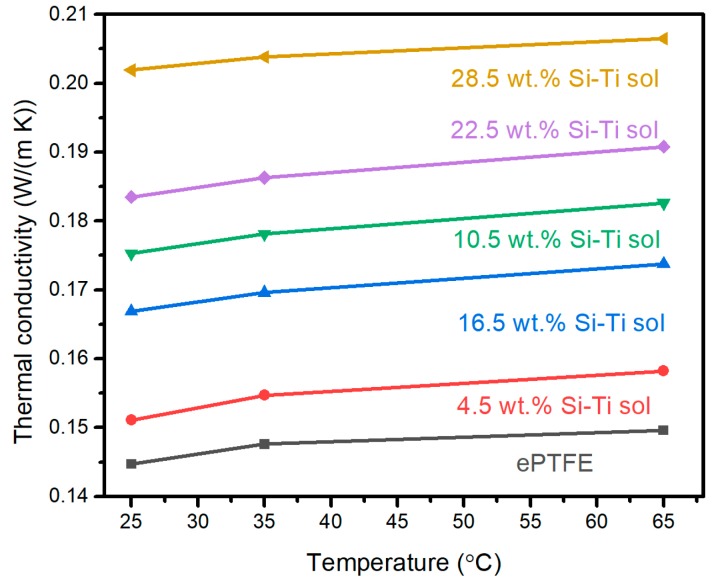
The thermal conductivity of MSQ aerogel-layer-coated ePTFE film with different Si-Ti sol contents.

Due to the small size of Si-Ti sol nanoparticles (about 4 nm), the pores of MSQ aerogel are filled with the cured Si-Ti sol. According to previous reports, MSQ aerogel exhibits a lower thermal conductivity because of the small fraction of solid silica and the unique pore size. However, pores filled with Si-Ti sol increase the solid silica rate and result in high thermal conductivity, as shown in Figure 7. With increasing temperature, the thermal conductivity of MSQ aerogel coating on ePTFE thin film increases, owing to the higher gas mean free path. The pristine ePTFE film shows the lowest thermal conductivity of 0.1447 W/(m·K) at 25 °C. After being coated with an MSQ aerogel layer, the thermal conductivity increases with increasing content of Si-Ti sol. As mentioned above, small nanoparticles in Si-Ti sol can fill the micropores between aerogel nanoparticles. With increasing content, the solid silica rate of the MSQ aerogel layer increases and more pores are filled, resulting in a gradual increase in thermal conductivity from 0.1551 W/(m·K) (4.5 wt.% Si-Ti sol at 25 °C) to 0.2019 W/(m·K) (28.5 wt.% Si-Ti sol at 25 °C). Additionally, for the coated ePTFE film with the best mechanical properties with 10.5 wt.% Si-Ti sol, the thermal conductivity increases from 0.1753 W/(m·K) at 25 °C to 0.1826 W/(m·K) at 65 °C. The high thermal conductivity compared with PTFE or polyvinyl chloride (PVC) is beneficial to decrease the cable temperature, especially for power cables.

**Figure 8:**
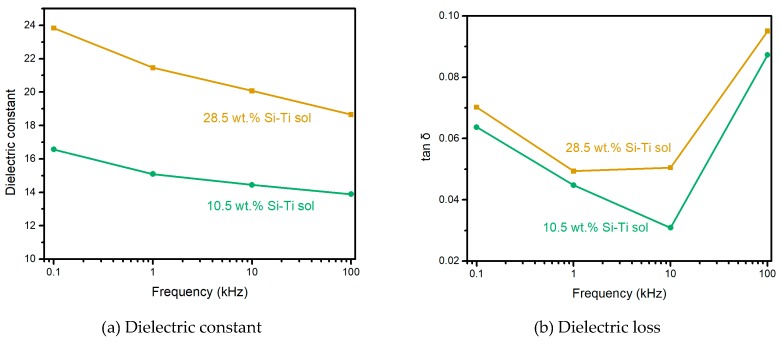
The dielectric constant (**a**) and dielectric loss (**b**) of ePTFE film coated by an MSQ aerogel layer with 10.5 wt.% and 28.5 wt.% Si-Ti sols.

Electrical properties are important for the application of the cable sheath. As mentioned above, an MSQ aerogel coating on ePTFE thin film with 16.5 wt.% Si-Ti sol as a binder shows the best mechanical property, and the one with 28.5 wt.% Si-Ti sol shows the best thermal conductivity. Figure 8 shows the dielectric constant and loss of the two coated ePTFE films. It can be observed that the MSQ aerogel coating on ePTFE thin film with 28.5 wt.% Si-Ti sol shows a higher dielectric constant and dielectric loss. As the frequency increases from 100 Hz to 100 kHz, the dielectric constant decreases from 23.8 to 18.7 for 28.5 wt.% Si-Ti sol and from 16.6 to 13.9 for 10.5 wt.% Si-Ti sol. The ePTFE film and MSQ aerogel show a lower dielectric constant, which indicates that the high dielectric constant of coated ePTFE thin film is caused by cured Si-Ti sol. For a cable sheath, the lower dielectric constant can promote the stability of cables, which shows that an aerogel coating layer with 10.5 wt.% Si-Ti sol and a dielectric loss of 0.06369 is the best. As is well-known, the introduced TiO_2_ exhibits a high dielectric constant of over 40. With the increase of Si-Ti sol content, the higher contact surface area decreases the carrier mobility, resulting in interfacial polarization, a high dielectric constant, and high dielectric loss compared with PTFE or PVC, which have a low dielectric constant. The MSQ aerogel coating layer enhances the electrical insulation property of ePTFE film, such that the breakdown voltage changes form lower than 500 V for pristine ePTFE thin film to 1020 V for an MSQ aerogel coating layer with 16.5 wt.% Si-Ti sol and even 1330 V for a coating layer with 28.5 wt.% Si-Ti sol. MSQ aerogel-coated ePTFE thin films with high insulation properties show great potential for application in cable sheaths.

## 3. Materials and Methods

### 3.1. Synthesis of the MSQ Aerogel Monolith

The MSQ aerogel monolith was synthesized by a sol-gel process followed by ambient drying in an oven at 40 °C for 12 h. For hydrolysis and polymerization, Methyltrimethoxysilane (MTMS, Aladdin, Shanghai, China, 98%) was added into a mix of hexadecyltrimethylammonium chloride (CTAC, Aladdin, 97%), methanol, and HCl solution, with vigorous stirring and an ice-bath for 30 min. Propylene oxide (PO, Sinopharm Chemical Reagent Co., Ltd., Shanghai, China, ≥99.5%) was then added as a gelation agent into the mixed solution and stirred for 2 min. The fabricated mix solution was closed and placed into an oven at 40 °C for the gelation process for about 40 min and a further aging process for 40 min. The gel was further solvent-exchanged with 2-propanol, hexamethyldisilane/n-heptane, and heptane (Sinopharm Chemical Reagent Co., Ltd., Shanghai, China, ≥99.5%) to eliminate CTAC and water. Finally, the solvent-exchanged gels were dried at 40 °C for 24 h to prepare the monolith of MSQ aerogel.

### 3.2. Fabrication of an MSQ Aerogel Coating Layer on ePTFE Thin Film

The MSQ aerogel powder was ball-milled for 8 h, and the aerogel powder was added to a mixture of Si-Ti sol as a binder and ethyl alcohol as a solvent. After 30 min of stirring, an alcohol-based aerogel slurry was prepared for coating. An automatic coating machine (FA 202D, Shanghai Xianpu Industrial Co., Ltd., Shanghai, China) equipped with a scraper was utilized to coat the slurry on ePTFE film. Finally, heat treatment at 170 °C for 30 min was carried out to cure the Si-Ti sol, and MSQ aerogel-coated ePTFE thin films were fabricated.

### 3.3. Characterization

The MSQ aerogel and coated ePTFE film were observed with an SU8010high resolution microscope with an accelerating voltage of 3 kV. The pore structures of the MSQ aerogel powder after ball-milling were measured by an N_2_ adsorption–desorption apparatus (BET, ASAP2020HD88, Micromeritics Instruments Corporation, Norcross, GA, USA), and the sample was degassed at 120 °C under vacuum before each N_2_ adsorption–desorption measurement. A Veeco instrument was utilized to produce atomic force microscopy (AFM) images. The mechanical properties were tested by a nanoelectromechanical universal testing machine (CMT4202). The thermal conductivity was obtained from a Hot Disk TPS 2500S. An LCR Bridge Meter was used to test the dielectric properties of MSQ aerogel coating on ePTFE thin film. The breakdown voltage was tested via a withstand voltage tester (CC2674-4, Nanjing Changchuang Science and Technology Ltd., Jiangsu, China).

## 4. Conclusions

MSQ aerogel-coated ePTFE thin films were fabricated successfully through a coating process for alcohol-based aerogel slurry containing only MSQ aerogel and Si-Ti sol, followed by heat treatment at 170 °C for 30 min. The MSQ aerogel synthesized via a sol-gel process exhibits a high specific surface area of 681.64 m^2^/g and an average pore diameter of 37 nm. The introduction of Si-Ti sol as a binder enhanced the combination between the MSQ aerogel layer and ePTFE film to eliminate the drop of aerogel powders and form a uniform aerogel layer without cracks. However, excess Si-Ti sol was found to fill the pores of the MSQ aerogel, resulting in poor elongation at break and high thermal conductivity. Finally, the ePTFE flexible thin film coated by an MSQ aerogel layer with 10.5 wt.% Si-Ti sol exhibits an elongation at break decrease of over 130%, a thermal conductivity of 0.1753 W/(m·K) at 25 °C, a dielectric constant of 16.5674, and a dielectric loss of 0.06369. The resultant ePTFE flexible thin film coated by an MSQ aerogel layer has high flexibility, uniformity, and integrity and a good electrical insulation property. It is a promising competitor for application in cable sheaths.
